# The pattern of lymph node metastasis in peripheral pulmonary nodules patients and risk prediction models

**DOI:** 10.3389/fsurg.2022.981313

**Published:** 2022-08-09

**Authors:** Lei Ke, Honghai Ma, Qingyi Zhang, Yiqing Wang, Pinghui Xia, Li Yu, Wang Lv, Jian Hu

**Affiliations:** Department of Thoracic Surgery, The First Affiliated Hospital, School of Medicine, Zhejiang University, Hangzhou, China

**Keywords:** peripheral pulmonary nodules (PPNs), lymph node, sentinel node (SN), risk prediction models, pattern of lymph node metastasis

## Abstract

**Background:**

For peripheral pulmonary nodules, the regularity of lymph node (LN) metastasis has not been studied. This study aimed to evaluate the metastasis pattern of intrapulmonary and relevant mediastinal lymph nodes in early-stage lung cancer, and further selected patients who were of low risk of LN metastasis as potential population to receive sub-lobectomy.

**Methods:**

This study prospectively included consecutive patients with peripheral clinical T1N0M0 disease who underwent complete resection with LN dissection or sampling from August 2014 to July 2015. The patients were followed up to 15, May 2021. Univariable or multivariable Logistic analysis was used to identify the risk factors. Models predicting LN metastasis risk were conducted. The area under the curve for the receiver operating characteristic curves was used to evaluate the diagnostic value. Disease-free survival and overall survival were compared between groups.

**Results:**

Finally, 201 patients were included in this study. For patients with negative tumor-bearing (TB) 13 and 14 station LNs, the positive rate of other lymph node stations was extremely low. Maximum CT value, pleural indentation and CEA level were risk factors for N1 station LNs metastasis. Besides, the factors above and lobulation sign were risk factors for skip metastasis beyond TB 13 and 14 station LNs. We constructed two scoring tables to predict N1 station metastasis and skip metastasis beyond TB 13 and 14 station. The AUC were 0·837 and 0·823, respectively. Based on the first table, 40·9% of patients suffered N1 station LNs metastasis and 27·3% had N2 disease in “high risk group” while the proportion was only 5·7% and 4·5% in “low risk group”. For patients with negative TB13 and TB14 station LNs, based on the latter table, 11·1% of patients had N1 stations LNs metastasis and 16·7% had pN2 disease in “high risk group” while only 2·3% patients in “low risk group” suffered this kind of metastasis.

**Conclusion:**

For peripheral pulmonary nodules patients, stations 13 and 14 LNs may be the sentinel nodes. For patients with low risk of N1 metastasis and skip metastasis, sub-lobar resection might be sufficient for those who were of negative TB 13 and 14 station LNs.

## Introduction

Globally, lung cancer is the second most common cancer, and about 2·2 million new cases of lung cancer were diagnosed in 2020 (1). It's also the leading cause of cancer-related death with an estimated 1·8 million deaths worldwide (1). Non-small cell lung cancer (NSCLC) occurs with the highest frequency in lung cancer (2). Low-radiation-dose computed tomography (low-dose CT) can improve the likelihood of detection of lung cancer at an earlier stage (3–5) and most lung cancer that can be detected by CT develops in the periphery of the lungs (6). Nodules that are present beyond the visualized segmental bronchi are usually called peripheral pulmonary nodules (7).

Surgical resection is considered the standard treatment for patients with stage I or II lung cancer (8) and recent guideline recommended anatomic lung resection (9). While Takahiro Mimae and Morihito Okada reported that in early-stage NSCLC, segmentectomy has an advantage over lobectomy, with a comparable curability to and less toxicity than lobectomy (10).

Lymph node metastasis is the most common and major metastatic pathway of NSCLC as well as the most important factor which affect staging and prognosis. Accurate evaluation of lymph node metastasis is of great significance for guiding surgical methods and adjuvant therapy. Lymph node metastasis in lung cancer is associated with lymphatic drainage. Pulmonary lymph first flows to the lymph nodes around the segmental bronchi, then back to the second hilum of the lung, and thence to hilar lymph nodes (11). It is generally recognized that lymph node metastasis of lung cancer is affected by many factors, such as the location, size, pathological type, and differentiation of the primary tumor (12). Intrapulmonary lymph nodes are cleaned with the resection of a pulmonary lobe. Compared to lobectomy, lymph node dissection in other segments can be skipped during segmentectomy. Thus, intrapulmonary lymph nodes dissection may be missed in those patients with peripheral pulmonary nodules and a high risk of intrapulmonary lymph nodes metastasis. It's necessary for us to distinguish which patients are at low risk of intrapulmonary lymph node metastasis that they may benefit from segmentectomy. Until now, there is a lack of studies like that.

We conducted this study to research metastasis of intrapulmonary and mediastinal lymph nodes in early-stage lung cancer. Besides, we attempted to screen out those who are at low risk of intrapulmonary and mediastinal lymph node metastasis, to evaluate less extent of pulmonary resection for this population of patients.

## Patients and methods

### Patient selection

This study was approved by the Medical Ethics Committee of the First Affiliated Hospital, School of Medicine, Zhejiang University, the reference number is 2013-255-1. Every individual participant had signed an informed consent form for participating in the study.

This prospective study initially included 331 consecutive patients with peripheral clinical T1N0M0 disease who underwent a complete resection in the department of thoracic, the First Affiliated Hospital from August 2014 to August 2015. Systematic mediastinal lymph node (LN) dissection or sampling was performed on each patient and intrapulmonary LNs (N1 LNs) were also systematically collected from the resected tissue samples by a trained thoracic surgeon cooperating with a pathologist. Systematic lymph node dissection (SLND), lobe-specific lymph node dissection (L-SLND) and selective lymph nodal sampling (SLNS) were conducted. Segmental bronchus and the intersegmental veins were carefully dissected and identified as segmental borders. The segmental nodes (station 13) and subsegmental nodes (station 14) were dissected and further divided as tumor-bearing (TB) or non-tumor-bearing (NTB) based on the location of the tumor.

Each patient enrolled in our study was followed up by telephone call and outpatient service every 3 months within the first year after the operation and every 6 months after the postoperative one year.

The exclusion criteria were as followed: (1) Patients have more than 1 lesion considered to be malignant; (2) patients with a history of another cancer or received any anti-tumor therapy preoperatively; (3) a selected lymph node biopsy was performed; (4) histological type was not invasive adenocarcinoma; (5) tumor invade all segments within a lobe; (6) patients with parietal pleura invasion; (7) Tumor-bearing station 13 and 14 LN were not evaluated; (8) loss to follow up; (9) suffered another malignancy during the follow-up procedure; (10) data was not complete (Supplementary Figure S1).

### Histological evaluation of the primary tumor and LNs

All tumors were staged according to the malignant tumor staging system in the eighth edition of the Union for International Cancer Control TNM Classification. Visceral pleural invasion (VPI) was defined as tumor cells invading beyond the elastic layer of the visceral pleura. If the invasion status was equivocal on hematoxylin-eosin–stained sections, an elastin stain was performed to confirm the presence or absence of VPI.

### Study endpoint

The outcomes of this study included overall survival (OS) and disease-free survival (DFS). The OS is the time from the operation to the death from any cause. DFS is the time from the operation to the first tumor recurrence/metastasis or death of the patients for any reason. The latest follow-up of the current study was performed on May 15, 2021.

### Statistical analysis

Categorical variables were compared using the *χ*2 test, while continuous variables were analyzed using the t-test, Mann-Whitney U test. Univariable or multivariable Logistic analysis was used to identify the risk factors. The area under the curve (AUC) and its standard error (SE) for the receiver operating characteristic (ROC) curves were used to evaluate the diagnostic value. All endpoints were estimated using the Kaplan-Meier (KM) method and compared by the log-rank test. Two-sided *p* values of <0·05 were considered statistically significant. All analyses were performed using SPSS 22·0 software (IBM, Armonk, NY), GraphPad Prism 7·0 software (GraphPad Software, La Jolla, Ca).

## Results

### Patient characteristics

Finally, 201 patients were included in this study. Most patients were female, younger than 65 years old with clinical T1b-1c disease. There were 14 (7·0%) patients who had pN1 disease and 19 (9·5%) participants suffered pN2 disease. The detailed characteristics of our study population were shown in Table 1.

**Table 1:** Demographics and clinicopathological characteristics of the study population (*n* = 201).

Characteristic	
Age (*n*, %)	
<65 years	149 (74·1)
≥65 years	52 (25·9)
Gender, (*n*, %)	
Male	70 (34·8)
Female	131 (65·2)
Smoking index, (*n*, %)	
<400	168 (83·6)
400–800	14 (7·0)
>800	19 (9·5)
COPD, (*n*, %)	
Yes	9 (4·5)
No	192 (95·5)
Diabetes, (*n*, %)	
Yes	52 (25·9)
No	149 (74·1)
cT-stage, (*n*, %)	
1a	15 (7·5)
1b	102 (50·7)
1c	84 (41·8)
Tumor consistency, (*n*, %)	
Puro GGO	45 (22·4)
Mixed GGO	96 (47·8)
Solid nodule	60 (29·9)
Spicule sign, (*n*, %)	
Negative	109 (54·2)
Positive	92 (45·8)
Lobulation sign, (*n*, %)	
Negative	141 (70·1)
Positive	60 (29·9)
Pleural indentation, (*n*, %)	
Negative	156 (77·6)
Positive	45 (22·4)
Surgical type, (*n*, %)	
Lobectomy	186 (92·5)
Segmentectomy	15 (7·5)
Lymph node dissection, (*n*, %)	
SLND	64 (31·8)
L-SLND	18 (9·0)
SLNS	119 (59·2)
pN-stage, (*n*, %)	
N0	168 (83·6)
N1	14 (7·0)
N2	19 (9·5)

COPD, chronic obstructive pulmonary disease; GGO, ground-glass opacity; SLND, systematic lymph node dissection; L-SLND, lobe-specific lymph node dissection; SLNS, selective lymph nodal sampling.

### Lymph node metastasis pattern in cT1N0M0 peripheral lung adenocarcinoma patients

Among all the patients, 33 (16·4%) patients had up-staging disease regardless of N1 or N2 lymph node metastasis. No lymph node metastasis was observed in patients with tumor diameters smaller than 1 centimeter.

**Figure 1:**
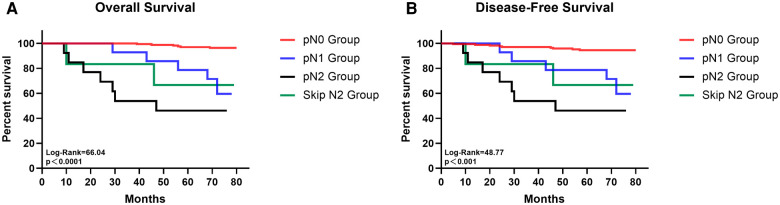
The survival curves of overall survival (**A**) and disease-free survival (**B**) for patients with pN0, pN1, pN2 and skip N2 disease.

18 (7·3%) patients had TB station 13 or 14 lymph node metastasis and among them 7 had only these two stations positive. For patients with negative TB 13 and 14 station lymph nodes, 2 (1·1%) cases had NTB station 13 and 14 lymph node metastasis and 14 patients had positive station 12 to N2 station lymph node. Among those N1 station lymph node metastasis patients, 71·4%(10/14) of them had only station 13 or 14 lymph node metastasis. Skip N2 metastasis was defined as the N2 station lymph nodes positive when all N1 station lymph nodes were negative. In our study, 6 (3·4%) cases showed skip N2 metastasis (Table 2). Survival analysis indicated that patients with pN0 disease had significantly better OS and DFS than pN1, general pN2 and skip pN2 disease while the survival outcomes of skip pN2 patients were better than general pN2 patients in both OS and DFS even though they were of the same pN stage (pN0 vs pN1 vs pN2 vs skip pN2: 79·03 vs 69·00 vs 47·92 vs 62·00 months for mean OS, *p* < 0·0001; 77·42 vs 66·71 vs 47·92 vs 62·00 months for mean DFS, *p *< 0·001) (Figure 1).

**Table 2:** Lymph node metastasis pattern for clinical stage IA (cT1N0M0) peripheral lung adenocarcinoma in left and right sides stratified by tumor size.

	Left lung				Right lung			
	Total (*n* = 89)	1 cm (*n* = 6)	2 cm (*n* = 46)	3 cm (*n* = 37)	Total (*n* = 112)	1 cm (*n* = 9)	2 cm (*n* = 57)	3 cm (*n* = 47)
Number of dissected LN, median(p25,p75)	18·0(13·0,23·0)	19·5(11·3,24·5)	17·0(13·0,22·3)	19·0(15·0,23·0)	20·0(15·3,24·8)	18·0(13·0,25·5)	19·5(15·3,24·0)	21·0(16·0,27·0)
Rate of up-staging (pN+), n (%)	16 (18·0)	0 (0·0)	8 (17·4)	8 (21·6)	17 (15·2)	0 (0·0)	4 (7·0)	13 (27·7)
Rate of only TB13 or TB14 LN metastasis, n (%)	3 (3·4)	0 (0·0)	2 (4·3)	1 (2·7)	4 (3·6)	0 (0·0)	1 (1·8)	3 (6·4)
Rate of TB13 or TB14 LN positive and other LN metastasis, n (%)	7 (7·9)	0 (0·0)	2 (4·3)	5 (13·5)	4 (3·6)	0 (0·0)	1 (1·8)	3 (6·4)
Rate of skip metastasis beyond TB 13 or TB14 LN, n (%)	6 (6·7)	0 (0·0)	4 (8·6)	2 (5·4)	9 (8·0)	0 (0·0)	2 (3·5)	7 (14·9)

CM, centimeter; LN, lymph node; SD, standard deviation; TB, tumor-bearing.

### Risk factors associated with N1 station lymph nodes metastasis

Totally, 27 (13·4%) patients suffered from N1 station lymph nodes metastasis. The factors that were significantly associated with the occurrence of N1 station lymph nodes metastasis were sizes of tumors, maximum CT value, the solid component of tumors, the occurrence of lobulation sign and pleural indentation, a rise of preoperative tumor markers, lower differentiation of tumors, VPI, solid, papillary or micropapillary growth component. Furthermore, multivariable regression analysis showed that maximum CT value, preoperative carcinoma embryonic antigen level, differentiation of tumors, VPI, and appearance of papillary growth component was an independent predictor of the metastasis of N1 station lymph nodes metastasis (Table 3).

**Table 3:** Regression analysis of the metastasis status in N1 lymph node.

Variable	Univariate analysis			Multivariate analysis		
	OR	95 CI	*p-*value	OR	95 CI	*p-*value
Age			* *			* *
<65 years	reference					
≥65 years	1·523	0·637–3·640	*0·344*			
Gender			* *			* *
Male	reference		* *			* *
Female	1·315	0·544–3·177	*0·543*			* *
Smoking index			* *			* *
None	reference		* *			* *
400–800	0·440	0·055–3·514	*0·439*			* *
>800	0·318	0·041–2·488	*0·275*			* *
Diameter of tumor			* *			* *
<2 cm	reference		* *	reference		* *
≥2 cm	2·715	1·174–6·280	*0·020*			*0·978*
Maximum CT value			* *			* *
<−75 Hu	reference		* *	reference		* *
≥−75 Hu	12·174	3·530–41·987	*<0·001*	13·126	2·681–64·255	*0·001*
Tumor consistency			* *			* *
Puro GGO	reference		* *	reference		* *
Mixed GGO	4·000	0·485–32·997	*0·198*			*0·717*
Solid nodule	18·857	2·409–147·595	*0·005*			*0·527*
Spicule sign			* *			* *
Negative	Reference		* *			* *
Positive	1·575	0·696–3·561	*0·275*			* *
Lobulation sign			* *			* *
Negative	reference		* *	reference		* *
Positive	3·182	1·389–7·287	*0·006*			*0·368*
Vacuole sign			* *			* *
Negative	reference		* *			* *
Positive	0·693	0·152–3·172	*0·637*			* *
Pleural indentation			* *			* *
Negative	Reference		* *	reference		* *
Positive	4·968	2·126–11·610	*<0·001*			*0·062*
CEA			* *			* *
≤5 ng/ml	Reference		* *	reference		* *
>5 ng/ml	5·804	2·433–13·842	*<0·001*	3·636	1·237–10·685	*0·019*
CA125			* *			* *
≤35 U/ml	Reference		* *	reference		* *
>35 U/ml	10·750	1·708–67·651	*0·011*			*0·309*
CA199			* *			* *
≤37 U/ml	Reference		* *	reference		* *
>37 U/ml	7·125	1·360–37·336	*0·020*			*0·150*
Differentiation			* *			* *
Low	Reference		* *	Reference		* *
Middle	0·084	0·030–0·239	*<0·001*	0·164	0·051–0·526	*0·002*
High	0·074	0·009–0·588	*0·014*	0·710	0·064–7·929	*0·781*
Visceral pleural invasion			* *			* *
No	Reference		* *	Reference		* *
Yes	5·863	2·483–13·845	*<0·001*	3·158	1·066–9·351	*0·038*
Invasion of bronchus			* *			* *
No	Reference		* *			* *
Yes	2·240	0·428–11·717	*0·339*			* *
Vascular cancer embolus			* *			* *
No	Reference		* *	reference		* *
Yes	9·913	2·085–47·125	*0·004*			*0·991*
Adherent growth component			* *			* *
No	Reference		* *	reference		* *
Yes	0·171	0·068–0·427	*<0·001*			*0·537*
Papillary growth component			* *			* *
No	Reference		* *	reference		* *
Yes	3·710	1·351–10·191	*0·011*	7·339	1·671–32·229	*0·008*
Micropapillary growth component			* *			* *
No	Reference		* *	reference		* *
Yes	3·538	1·215–10·305	*0·020*			*0·814*
Acinar growth component			* *			* *
No	Reference		* *			* *
Yes	1·356	0·542–3·394	*0·515*			* *
Solid growth component			* *			* *
No	Reference		* *	reference		* *
Yes	4·097	1·705–9·847	*0·002*			*0·840*
EGFR mutation			* *			* *
Wild type	Reference		* *			* *
Mutated	0·851	0·284–2·552	*0·774*			* *

CEA, carcinoma embryonic antigen; CI, confidence interval; CT, computed tomography; EGFR, epidermal growth factor receptor; GGO, ground glass opacity; Hu, Hounsfield unit; mL, milliliter; ng, nanogram; OR, odds ratio.

**Figure 2:**
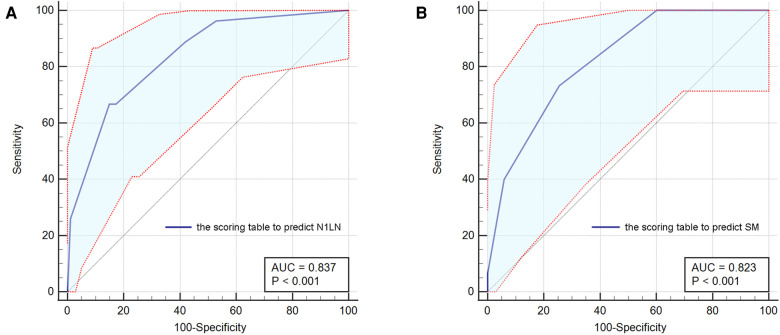
The ROC curves for the scoring table to predict N1LN (**A**) and the scoring table to predict SM (**B**). ROC, receiver operating characteristic; N1LN, N1 station lymph nodes metastasis; SM, skip metastasis beyond TB 13 and 14 station lymph nodes.

**Figure 3:**
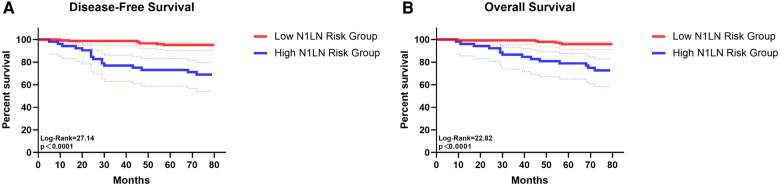
The survival curves of disease-free survival (**A**) and overall survival (**B**) for patients in low N1LN risk group and in high N1LN risk group.

To use preoperative characteristics to predict the metastasis of N1 station lymph nodes, extra regression analysis with only preoperative factors was performed, the results were shown in Supplementary Table S1. Based on the results of the regression analysis, an index that predicts which we called “N1LN” was formulated (Supplementary Table S2) The total score of the “N1LN” index was 10 points and drawing a ROC curve, the AUC of this index was 0·837 (Figure 2A). The cutoff value was 6·5 which was calculated by the ROC curve. Based on the cutoff value, patients were divided into “High N1LN risk Group” and “Low N1LN risk Group”. In the “High N1LN risk Group”, 40·9% (18/44) of patients suffered N1 station lymph nodes metastasis and 27·3% (12/44) had N2 disease while the proportion was only 5·7% (9/157) and 4·5% (7/157) in the “Low N1LN risk Group”, and the differences were of statistical significance (*p* < 0·001; *p* < 0·001, respectively). Moreover, the patients in the “High N1LN risk Group” had significantly worse prognosis in terms of not only OS (mean OS: 68·14 vs 78·55 months, log-rank = 22·82, *p* < 0·0001) but also DFS (mean DFS: 63·84 vs 78·05 months, log-rank = 27·14, *p* < 0·0001) (Figures 3A,B).

### Regression analysis for preoperatively predictive factors of skip metastasis beyond TB 13 and TB 14 station lymph nodes

Several preoperative parameters were enrolled in the univariable regression analysis. The diameter of tumors, maximum CT value, tumor consistency, the appearance of spicule sign, lobulation sign or pleural indentation, preoperative CEA, CA125, and CA199 were identified to be associated with the metastasis of other lymph node stations when TB 13 and TB 14 LNs were negative. And the results of the multivariable analysis indicated that maximum CT value larger than −75 Hu, the appearance of lobulation sign and pleural indentation, and the rise of preoperative CEA levels were independently associated with skip metastasis beyond TB 13 and TB 14 station lymph nodes (Table 4).

**Table 4:** Preoperative factors predicting skip metastasis beyond tumor-bearing 13 and tumor-bearing 14 lymph node.

Variable	Univariate analysis			Multivariate analysis		
	OR	95 CI	*p-*value	OR	95 CI	*p*-value
Age			* *			* *
<65 years	reference					
≥65 years	1·091	0·330–3·609	0·887			
Gender			* *			* *
Male	reference		* *			* *
Female	0·603	0·208–1·745	*0·350*			* *
Smoking index			* *			* *
None	reference		* *			* *
400–800	2·331	0·458–11·857	*0·308*			* *
>800	1·602	0·326–7·880	*0·562*			* *
Diameter of tumor			* *			* *
<2 cm	reference		* *	reference		* *
≥2 cm	2·500	0·850–7·356	*0·096*	0·981		* *
Maximum CT value			* *			* *
<−75 Hu	reference		* *	reference		* *
≥−75 Hu	4·358	1·331–14·263	*0·015*	3·565	1·010–12·589	*0·048*
Tumor consistency			* *			* *
Puro GGO	reference		* *	reference		* *
Mixed GGO	2·966	0·346–25·415	*0·321*	0·707		* *
Solid nodule	9·053	1·082–75·733	*0·042*	0·386		* *
Spicule sign			* *			* *
Negative	Reference		* *	Reference		* *
Positive	2·603	0·853–7·945	*0·093*			*0·832*
Lobulation sign			* *			* *
Negative	reference		* *	reference		* *
Positive	4·800	1·610–14·309	*0·005*	3·890	1·201–12·602	*0·024*
Vacuole sign			* *			* *
Negative	reference		* *			* *
Positive	0·595	0·074–4·797	*0·626*			* *
Pleural indentation			* *			* *
Negative	Reference		* *	reference		* *
Positive	4·194	1·409–12·480	*0·010*	3·972	1·216–12·975	*0·022*
CEA			* *			* *
≤5 ng/ml	Reference		* *	reference		* *
>5 ng/mL	4·933	1·588–15·327	*0·006*	3·634	1·059–12·464	*0·040*
CA125			* *			* *
≤35 U/ml	Reference		* *	reference		* *
>35 U/ml	N/A		* *			* *
CA199			* *			* *
≤37 U/ml	Reference		* *	reference		* *
>37 U/ml	N/A		* *			* *

CEA, carcinoma embryonic antigen; CI, confidence interval; CT, computed tomography; GGO, ground glass opacity; Hu, Hounsfield unit; mL, milliliter; ng, nanogram; OR, odds ratio.

Based on the results, we further constructed another scoring table (Supplementary Table S3) to predict the possibility of skip metastasis beyond TB 13 and 14 station lymph nodes which combined the aforementioned 4 independent risk factors and was called the “SM” index. The score was significantly correlated with the risk of skim metastasis beyond TB segmental or sub-segmental lymph nodes, and the AUC was 0·823 with the cut-off value of 3·75. For patients with negative TB13 and TB14 station lymph nodes, each of them was divided into “High SM risk Group” and “Low SM risk Group” according to the cut-off value.

**Figure 4:**
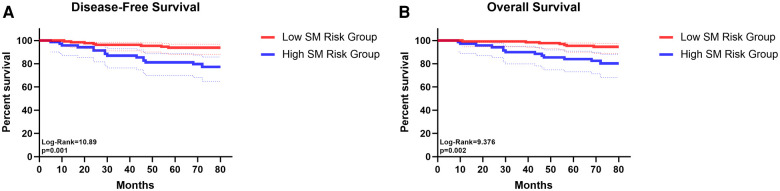
The survival curves of disease-free survival (**A**) and overall survival (**B**) for patients in low SM risk group and in high SM risk group.

Compared with the “Low SM risk Group”, the “High SM risk Group” harvested similar numbers of N1 and N2 station lymph nodes. However, in the “High SM risk Group”, 11·1% (6/54) of patients had N1 stations lymph nodes metastasis and 16·7% (9/54) had pN2 disease when the TB 13 and 14 station lymph nodes were negative while only 2·3% (3/129) patients in the “Low SM risk Group” suffered this kind of metastasis and the differences were of statistic significance (*p* = 0·033; *p* < 0·001, respectively) (Supplementary Table S4). The detailed lymph node metastasis status of each station between the high and low risk of “SM” index was shown in Supplementary Table S4. The K-M survival analysis and log-rank comparison revealed that the “Low SM risk Group” had better OS (mean OS: 78·23 vs 71·81 months, log-rank = 9·376, *p* = 0·002) and DFS (mean DFS: 77·02 vs 69·76 months, log-rank = 10·89, *p* = 0·001) than the “High SM risk Group” (Figures 4A,B).

## Discussion

Lobectomy had been generally accepted as a standard cure for NSCLC, a few authors had doubts as to whether lobectomy is necessary for treatment of small lesions (13). Hisashi Saji et al suggested that for patients with small-sized (≤2 cm, consolidation-to-tumor ratio >0·5) peripheral NSCLC, segmentectomy should be the standard surgical procedure instead of lobectomy (14). However, a few potential high-risk lymph nodes may be ignored during segmentectomy. Therefore, it's of great significance to determine the risk factors of lymph nodes metastasis, and those peripheral pulmonary nodules patients at low risk of intrapulmonary lymph nodes metastasis may be proper to undergo segmentectomy.

The aim of this study was to investigate the pattern of intrapulmonary lymph nodes metastasis and corresponding mediastinal lymph node metastases in patients with peripheral NSCLC. And based on the law that pulmonary lymph first flows to the lymph nodes around the segmental bronchi, then back to the second hilum of the lung, and thence to hilar lymph nodes (11), we try to determine whether TB station 13 or 14 lymph node metastasis can be a predictor for other lymph nodes metastasis. Then we can construct a model to distinguish patients at high or low risk of lymph node metastasis and discover the best surgical method and lymph node dissection. This study suggests that the cervical node involvement of lung adenocarcinoma is spread mostly orderly, and the incidence of skip metastasis is low. Certainly, there are a small number of skip pN2 patients, but their survival outcomes were better than general pN2 patients in both OS and DFS.

Stiles et al. compared lobectomy and sublobar resections on the basis of the extent of LN dissection in stage I NSCLC patients (15). When patients with at least 1 lymph node removed were examined, the survival rate of sublobar resections was lower than lobectomy (15). While when patients with ≥9 lymph nodes were examined, the overall survival and cancer-specific survival seemed to be similar (15). These findings show that only enough LN assessment is performed, might the oncological outcomes of sublobar resections be as effective as those of lobectomy (15). Lymph node metastasis is an important factor in treatment decisions. We can hardly find prospective and large-sample studies on clinical data of intrapulmonary lymph nodes especially stations 13 and 14 (16). In our study, among those N1 station lymph node metastasis patients, most of them had only station 13 or 14 lymph node metastasis. So we think it's necessary to perform station 13 and 14 lymph node dissection. However, as far as I know, many surgeons didn't dissect station 13 and 14 lymph nodes. Based on the pattern of lymph node metastasis, we constructed a table to evaluate N1 Station lymph node metastasis. Additionally, we found that patients with negative 13 and 14 station lymph nodes were less likely to have other station lymph nodes positive, which indicated that maybe they can act as the sentinel nodes. Further, we constructed another table to predict skip metastasis beyond TB 13 and TB 14 station lymph nodes. With the help of the tables, we can differentiate the high risk of lymph node metastasis from the low risk, and then we can point out which patient can take less extent of pulmonary resection. We can see significant differences in overall survival and disease-free survival between high-risk and low-risk groups.

Our tables consisted of maximum CT value, pleural indentation, lobulation sign, and CEA level. Preoperative imaging CT or PET-CT has limited ability to detect lymph nodes (17), thus people turn to research serum biomarkers. Carcinoembryonic antigen (CEA) is the most commonly used one in tumor markers (18–21), it was an important predictor for lymph node metastasis (22). In lung adenocarcinoma, the expression of CEA was correlated with lymph node metastasis (23). Ding et al.'s study showed that lobulated margins were associated with invasiveness (24). In pure GGNs, invasive pulmonary adenocarcinoma was more lobulated than preinvasive lesions (25). In the comparison of CT findings between invasive tumor and noninvasive tumor, they showed differences in terms of pleural indentation and lobulation (26). Ma et al. also found that the 5-year survival rate of patients with slick margin was a little higher than those with lobulation sign (27). They indicated that pleura indentation was one of the risk factors for poor prognosis of stage I NSCLC (27). Mean CT value was significantly associated with invasive extent (28). Likewise, maximum CT value was also a significant predictor of histologic invasiveness (29). The invasive component of pure GGO was present in the site with high CT value (30). In our study, multivariate analysis revealed that lower CT value, lower CEA level, negative pleural indentation and nonlobulated sign were important differentiators of low lymph nodes metastasis risk group (*p* < 0·01), with excellent differentiating accuracy (area under ROC curve, 0·823). By studying the pattern of intrapulmonary and mediastinal lymph nodes metastasis and finding the risk factors for lymph nodes metastasis, we tried to point out which patient can take less extent of pulmonary resection.

This is the first study that constructed two scoring tables to evaluate N1 station lymph nodes metastasis and predict skip metastasis beyond TB 13 and TB 14 station lymph nodes. With the two tables, doctors can find out low-risk patients and make optimal surgery plan for them. There are several limitations of this study. First, it was conducted at a single center. Second, the number of the sample may be not enough to support a strong level of evidence of its clinical application. Further multicenter studies and more cases are therefore needed to verify these results. Third, we didn't compare segmentectomy and lobectomy in the “Low SM risk Group”. Further study is needed to determine whether segmentectomy can be a perfect substitute for lobectomy in low lymph nodes metastasis risk group.

## Conclusion

In conclusion, different clinical features lead to the different probability of lymph node metastasis. For patients with peripheral pulmonary nodules, stations 13 and 14 lymph nodes may be the sentinel nodes. Therefore, for patients with high risk of N1 metastasis and skip metastasis, lobectomy is appropriate for those who were of positive TB 13 and 14 station lymph nodes. As for patients in the low-risk group, those who have negative TB 13 and 14 station lymph nodes may be the potential population who are proper to take sublobar resection. In addition, station 13 and 14 lymph node dissection should be performed in both lobectomy and sublobar resection.

## Data Availability

The raw data supporting the conclusions of this article will be made available by the authors, without undue reservation.
