# Supplementary figures and images for: The pattern of lymph node metastasis in peripheral pulmonary nodules patients and risk prediction models
Source: Front Surg. 2022 Aug 9;9:981313. doi: 10.3389/fsurg.2022.981313 (PMC9395917; doi:10.3389/fsurg.2022.981313)

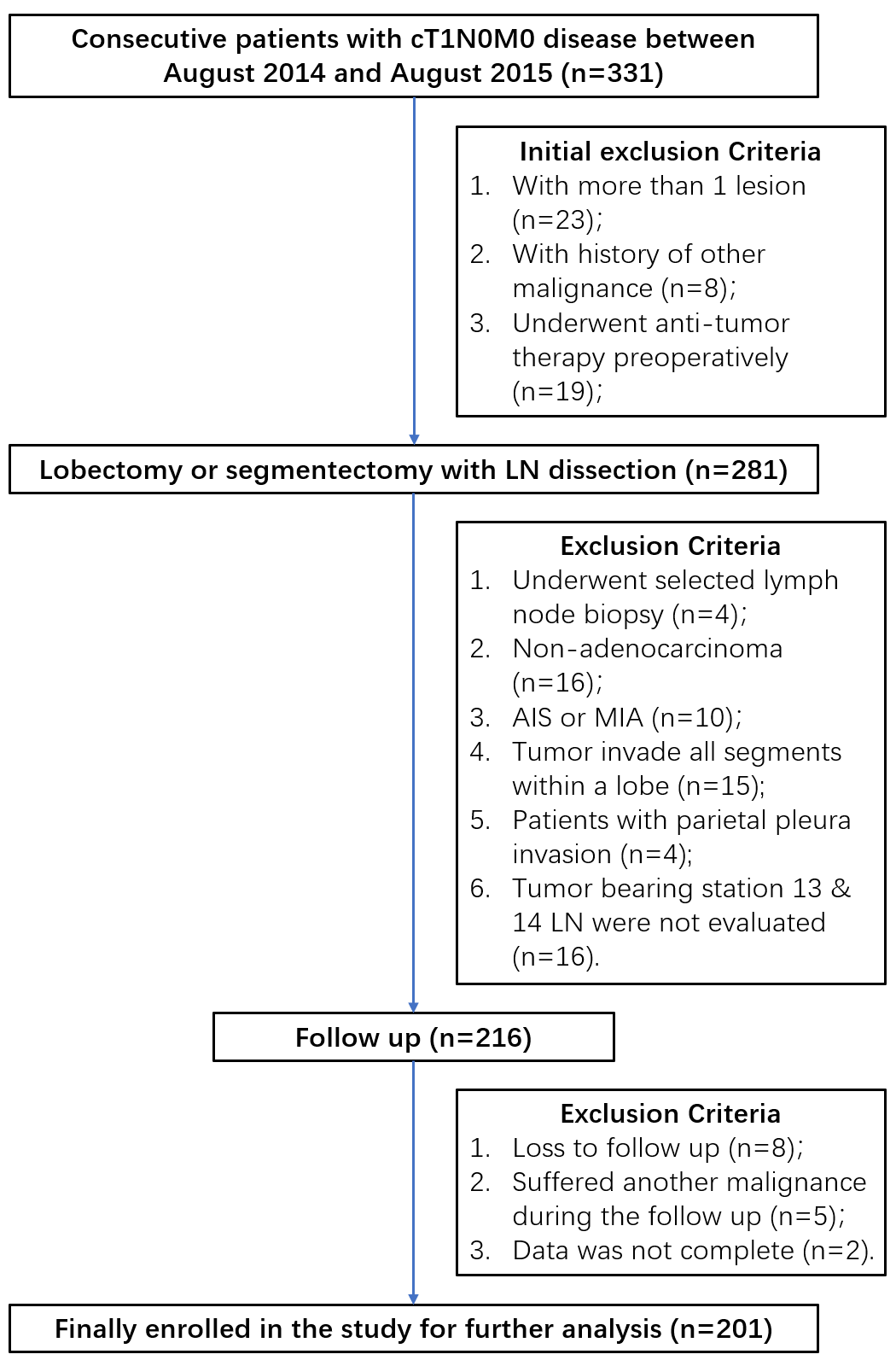

Supplement: Supplementary file 1 [file Image_1_v1.tif]
