# Supplementary material for: The pattern of lymph node metastasis in peripheral pulmonary nodules patients and risk prediction models
Source: Front Surg. 2022 Aug 9;9:981313. doi: 10.3389/fsurg.2022.981313 (PMC9395917; doi:10.3389/fsurg.2022.981313)
Supplement: Supplementary file 2 [file Table_1_v2.docx]

**Supplemental Table 1. Preoperative factors predicting N1 station lymph node metastasis.**

| **Variable** | **Univariate analysis** | | | **Multivariate analysis** | | |
| --- | --- | --- | --- | --- | --- | --- |
|  | **OR** | **95 CI** | **P value** | **OR** | **95 CI** | **P value** |
| **Age** |  |  |  |  |  |  |
| <65 years | reference |  |  |  |  |  |
| ≥65 years | 1.523 | 0.637-3.640 | 0.344 |  |  |  |
| **Gender** |  |  |  |  |  |  |
| Male | reference |  |  |  |  |  |
| Female | 1.315 | 0.544-3.177 | *0.543* |  |  |  |
| **Smoking index** |  |  |  |  |  |  |
| None | reference |  |  |  |  |  |
| 400-800 | 0.440 | 0.055-3.514 | *0.439* |  |  |  |
| >800 | 0.318 | 0.041-2.488 | *0.275* |  |  |  |
| **Diameter of tumor** |  |  |  |  |  |  |
| <2cm | reference |  |  | reference |  |  |
| ≥2cm | 2.715 | 1.174-6.280 | *0.020* |  |  | *0.959* |
| **Maximum CT value** |  |  |  |  |  |  |
| <-75 Hu | reference |  |  | reference |  |  |
| ≥-75 Hu | 12.174 | 3.530-41.987 | *<0.001* | 9.484 | 2.639-34.085 | *0.001* |
| **Tumor consistency** |  |  |  |  |  |  |
| Puro GGO | reference |  |  | reference |  |  |
| Mixed GGO | 4.000 | 0.485-32.997 | *0.198* |  |  | *0.105* |
| Solid nodule | 18.857 | 2.409-147.595 | *0.005* |  |  | *0.061* |
| **Spicule sign** |  |  |  |  |  |  |
| Negative | Reference |  |  |  |  |  |
| Positive | 1.575 | 0.696-3.561 | *0.275* |  |  |  |
| **Lobulation sign** |  |  |  |  |  |  |
| Negative | reference |  |  | reference |  |  |
| Positive | 3.182 | 1.389-7.287 | *0.006* |  |  | *0.095* |
| **Vacuole sign** |  |  |  |  |  |  |
| Negative | reference |  |  |  |  |  |
| Positive | 0.693 | 0.152-3.172 | *0.637* |  |  |  |
| **Pleural indentation** |  |  |  |  |  |  |
| Negative | Reference |  |  | reference |  |  |
| Positive | 4.968 | 2.126-11.610 | *<0.001* | 3.645 | 1.418-9.369 | *0.007* |
| **CEA** |  |  |  |  |  |  |
| ≤ 5ng/mL | Reference |  |  | reference |  |  |
| > 5ng/mL | 5.804 | 2.433-13.842 | *<0.001* | 4.458 | 1.696-11.716 | *0.002* |
| **CA125** |  |  |  |  |  |  |
| ≤ 35U/mL | Reference |  |  | reference |  |  |
| > 35U/mL | 10.750 | 1.708-67.651 | *0.011* |  |  | *0.089* |
| **CA199** |  |  |  |  |  |  |
| ≤ 37U/mL | Reference |  |  | reference |  |  |
| > 37U/mL | 7.125 | 1.360-37.336 | *0.020* |  |  | *0.112* |
| *CEA,* carcinoma embryonic antigen; *CI*, confidence interval; *CT,* computed tomography; *GGO,* ground glass opacity; *Hu,* Hounsfield unit*; mL,* milliliter; *ng,* nanogram; *OR*, odds ratio. | | | | | | |
