# Supplementary material for: The pattern of lymph node metastasis in peripheral pulmonary nodules patients and risk prediction models
Source: Front Surg. 2022 Aug 9;9:981313. doi: 10.3389/fsurg.2022.981313 (PMC9395917; doi:10.3389/fsurg.2022.981313)
Supplement: Supplementary file 4 [file Table_3_v2.docx]

**Supplemental Table 3. Scoring table for predicting skip metastasis beyond tumor-bearing 13 or tumor-bearing 14 lymph node.**

| **Index** | **Status** | **Score** |
| --- | --- | --- |
| Maximum CT value | <-75 Hu | 0 |
|  | ≥-75 Hu | 2.5 |
| Pleural indentation | Negative | 0 |
|  | Positive | 2.5 |
| Lobulation sign | Negative | 0 |
|  | Positive | 2.5 |
| CEA level | ≤ 5ng/mL | 0 |
|  | > 5ng/mL | 2.5 |
| *CEA,* carcinoma embryonic antigen; *CT,* computed tomography; *Hu,* Hounsfield unit*; mL,* milliliter; *ng,* nanogram. | | |
|  |  |  |
