# Supplementary material for: The pattern of lymph node metastasis in peripheral pulmonary nodules patients and risk prediction models
Source: Front Surg. 2022 Aug 9;9:981313. doi: 10.3389/fsurg.2022.981313 (PMC9395917; doi:10.3389/fsurg.2022.981313)
Supplement: Supplementary file 5 [file Table_4_v2.docx]

**Supplemental Table 4. Lymph node metastasis status between high and low risk of “SM” index when tumor-bearing 13 and tumor-bearing 14 station lymph node are negative.**

| **Variable** | **SM index group** | | **p** |
| --- | --- | --- | --- |
|  | **Low SM risk group (n=129)** | **High SM risk group (n=54)** |  |
| N1 station, n (%) |  |  | 0.033 |
| Negative | 126 (97.7) | 48 (88.9) |  |
| Positive | 3 (2.3) | 6 (11.1) |  |
| N2 station, n (%) |  |  | 0.001 |
| Negative | 126 (97.7) | 45 (83.3) |  |
| Positive | 3 (2.3) | 9 (16.7) |  |
| 2R, n (%)* |  |  | 0.260 |
| Negative | 77 (100.0) | 26 (96.3) |  |
| Positive | 0 (0.0) | 1 (3.7) |  |
| 3, n (%)* |  |  | 0.260 |
| Negative | 77 (100.0) | 26 (96.3) |  |
| Positive | 0 (0.0) | 1 (3.7) |  |
| 4R, n (%)* |  |  | 0.004 |
| Negative | 77 (100.0) | 23 (85.2) |  |
| Positive | 0 (0.0) | 4 (14.8) |  |
| 4L, n (%)* |  |  | 1.000 |
| Negative | 52 (100.0) | 27 (100.0) |  |
| Positive | 0 (0.0) | 0 (0.0) |  |
| 5, n (%)* |  |  | 0.603 |
| Negative | 50 (96.2) | 25 (92.6) |  |
| Positive | 2 (3.8) | 2 (7.4) |  |
| 6, n (%)* |  |  | 1.000 |
| Negative | 51 (98.1) | 27 (100.0) |  |
| Positive | 1 (1.9) | 0 (0.0) |  |
| 7, n (%) |  |  | 0.044 |
| Negative | 128 (99.2) | 51 (94.4) |  |
| Positive | 1 (0.8) | 3 (5.6) |  |
| 8, n (%) |  |  | 1.000 |
| Negative | 129 (100.0) | 54 (100.0) |  |
| Positive | 0 (0.0) | 0 (0.0) |  |
| 9, n (%) |  |  | 1.000 |
| Negative | 129 (100.0) | 54 (100.0) |  |
| Positive | 0 (0.0) | 0 (0.0) |  |
| 10, n (%) |  |  | 1.000 |
| Negative | 129 (100.0) | 54 (100.0) |  |
| Positive | 0 (0.0) | 0 (0.0) |  |
| 11, n (%) |  |  | 0.027 |
| Negative | 128 (99.2) | 50 (92.6) |  |
| Positive | 1 (0.8) | 4 (7.4) |  |
| 12, n (%) |  |  | 0.208 |
| Negative | 128 (99.2) | 52 (96.3) |  |
| Positive | 1 (0.8) | 2 (3.7) |  |
| 13NTB, n (%) |  |  | 0.295 |
| Negative | 129 (100.0) | 53 (98.1) |  |
| Positive | 0 (0.0) | 1 (1.9) |  |
| 14NTB, n (%) |  |  | 1.000 |
| Negative | 128 (99.2) | 54 (100.0) |  |
| Positive | 1 (0.8) | 0 (0.0) |  |
| Number of N1 LN dissected |  |  | 0.588 |
|  | 11.12±5.12 | 10.65±6.02 |  |
| Number of N1 LN metastasis |  |  | 0.033 |
|  | 0.04±0.291 | 0.17±0.505 |  |
| Number of N2 LN dissected |  |  | 0.873 |
|  | 8.91±5.28 | 9.06±5.69 |  |
| Number of N2 LN metastasis |  |  | 0.002 |
|  | 0.03±0.21 | 0.46±1.50 |  |
| *LN,* lymph node; *NTB,* non-tumor bearing. | | | |

* 2R\3\4R only for tumor located in the right side and 4L\5\6 only for tumor located in the left side.
